# Different Characteristics in Gut Microbiome between Advanced Adenoma Patients and Colorectal Cancer Patients by Metagenomic Analysis

**DOI:** 10.1128/spectrum.01593-22

**Published:** 2022-12-01

**Authors:** Shuwen Han, Jing Zhuang, Yuefen Pan, Wei Wu, Kefeng Ding

**Affiliations:** a Department of Colorectal Surgery and Oncology, Key Laboratory of Cancer Prevention and Intervention, Ministry of Education, Zhejiang Provincial Clinical Research Center for Cancer, The Second Affiliated Hospital, Zhejiang University School of Medicine, Hangzhou, Zhejiang, China; b Cancer Center, Zhejiang University, Hangzhou, Zhejiang, China; c Department of Medical Oncology, Huzhou Central Hospital, Huzhou, Zhejiang, China; Iowa State University

**Keywords:** colorectal cancer, metagenomic sequencing, gut microbiome, artificial intelligence, SNP

## Abstract

The occurrence and development of colorectal cancer (CRC) and advanced adenoma (AA) are closely related to the gut microbiome, and AA has a high cancerization progression rate to CRC. Current studies have revealed that bacteriological analysis cannot identify CRC from AA. The objective was to explore microbial targets that could identify CRC and AA from a microecological perspective and to figure out the best way to identify CRC based on fecal microbes. The metagenomic sequencing data were used to describe the gut microbiome profile and analyze the differences between microbial abundance and microbial single nucleotide polymorphism (SNP) characteristics in AA and CRC patients. It was found that there were no significant differences in the diversity between the two groups. The abundance of bacteria (e.g., *Firmicutes*, *Clostridia*, and *Blautia*), fungi (*Hypocreales*), archaea (*Methanosarcina*, *Methanoculleus*, and *Methanolacinia*), and viruses (*Alphacoronavirus*, *Sinsheimervirus*, and *Gammaretrovirus*) differed between AA and CRC patients. Multiple machine-learning algorithms were used to establish prediction models, aiming to identify CRC and AA. The accuracy of the random forest (RF) model based on the gut microbiome was 86.54%. Nevertheless, the accuracy of SNP was 92.31% in identifying CRC from AA. In conclusion, using microbial SNP was the best method to identify CRC, it was superior to using the gut microbiome, and it could provide new targets for CRC screening.

**IMPORTANCE** There are differences in characteristic microorganisms between AA and CRC. However, current studies have indicated that bacteriological analysis cannot identify CC from AA, and thus, we wondered if there were some other targets that could be used to identify CRC from AA in the gut microbiome. The differences of SNPs in the gut microbiota of intraindividuals were significantly smaller than those of interindividuals. In addition, compared with intestinal microbes, SNP was less affected by time with certain stability. It was discovered that microbial SNP was better than the gut microbiome for identifying CRC from AA. Therefore, screening characteristic microbial SNP could provide a new research direction for identifying CRC from AA.

## INTRODUCTION

In 2020, the incidence of colorectal cancer (CRC), one of the most common malignant tumors, in the world was 10.6% in males and 9.4% in females, and its mortality was 9.3% in males and 9.5% in females (1). Although many studies have shown that CRC appears to be related to changes in the gut microbiome, the pathogenesis of CRC is still unclear (2–5). As the impacts of the gut microbiome on metabolism and disease have been uncovered, the relationship between diet, the gut microbiome, and CRC has emerged (5). Advanced adenoma (AA) is one kind of adenoma (villous or mixed), with a diameter over 1 cm, and it is commonly accompanied with one of the three adenomas with moderate to severe dysplasia. As AA is a precancerous lesion of CRC, early detection and treatment of AA can reduce the morbidity and mortality of CRC (6, 7). In this present study, stool samples were collected, and the gut microbes of AA patients and CRC patients were analyzed to look for different microbial markers.

As a fecal storage organ, the gut is home to the largest microbial community of human body. The total number of bacteria in gut reached 10^14^, accounting for 98.8% of the total microbes of the human body (8). The gut microbiome, composed of gut bacteria, fungi, archaea, and viruses, is an independent and complex microecosystem, in which competition, predation, symbiosis, cooperation, and other interactions could be detected (9, 10). Under normal circumstances, the interaction of gut microorganisms is in equilibrium. Once an imbalance of gut microorganisms occurs, gut microecological balance would be broken, and gut microorganisms can induce CRC. Meanwhile, gut microorganisms can induce the occurrence of gut cancer through immune regulation, gene integration, inflammation, and other ways (11).

Previous research reported that AA includes some characteristic microorganisms such as *Fusobacterium* and Bacillus fragilis, while CRC includes some characteristic microorganisms such as *Parvimonas*, *Gemella*, *and Leptotrichia*. There are differences in characteristic microorganisms between AA and CRC (12). However, Xiao et al. (13) analyzed bacterial DNA in peripheral blood, which was different from that of previous studies, and no significant differences between AA patients and CRC patients were found. Therefore, we wondered if there were some other targets that can be used to identify AA and CRC in the gut microbiome.

Currently, the characteristic viruses associated with CRC, including coxsackievirus, adenovirus, human cytomegalovirus, human papillomavirus, African lymphocytomavirus, poliovirus, and hepatitis B virus, have been screened (14). Bacteriophages, as the most common and widely distributed group of viruses, can not only cure bacterial infections effectively but also activate immune responses and transform the tumor microenvironment into an environment that is conducive to anticancer treatment (4, 15–17). Considered together, this information suggests that bacteriophage may be a potential target to identify AA and CRC.

Single nucleotide polymorphism (SNP) is a genetic variation in DNA sequences caused by variations in a single nucleotide, and it is the most common form of human heritable variation, accounting for more than 90% of all known polymorphisms (18, 19). Since the differences in gene sequences or even differences in single bases lead to changes in gene function, metagenomics is not limited to the analysis of species and gene abundance but associated with changes in genes, such as single base mutations, insertions, deletions, and structural changes. A SNP associated with the gut microbiome may lead to intestinal diseases. For example, Zou et al. (20) found that the SNP of a bacterial immune suppression gene, *blc*, disrupts gut lysophospholipid homeostasis and induces inflammation through disrupting the epithelial barrier. In addition, Chen et al. (21) compared the SNP in gut microbes of the same individual and different individuals 4 years ago and now and found that the differences of SNPs in the gut microbes of the same individuals were significantly smaller than those between different individuals. Moreover, individual differences in gut microbiota structure could still be detected after a 4-year interval, which proved that SNP has certain stability. Therefore, screening characteristic microbial SNPs can provide a new research direction for screening CRC from AA.

A variety of CRC screening kits such as the LifeKit Prevent of Prescient Metabiomics based on intestinal fecal microbiome tests have been developed. These bowel cancer early-screening products were used to differentiate the bowel disease group and the normal group, while their accuracy in differentiating benign and malignant tumors, such as AA and CRC, is still unclear. Stool, a sample for colon cancer screening, is noninvasive and easy to obtain. Currently, various kits for CRC screening are used to detect microorganisms and genes. However, there is a lack of theoretical support for selecting the best indicators for CRC screening. Microbiome data in feces and microbial-related SNP data were compared to find the best method and optimal target for stool samples for CRC screening, aiming to provide data support for stool samples.

Although metagenomic studies of SNPs have been reported, there were relatively few studies on SNPs in gut microbes. This study compared the genes in stool samples of AA patients and CRC patients through metagenomic sequencing and analyzed differential genes of the gut microbiome between the two types of patients. The abundance and diversity of gut microbes in patients with colorectal polyps and patients with CRC were described and compared. Furthermore, SNPs in this genome in each fecal microbiome were identified by mapping the metagenomic sequences to a unified human gastrointestinal genome (UHGG) collection. Moreover, the best prediction model was constructed to distinguish AA from CRC based on SNPs.

The study aimed to elucidate the differences in the gut microbiome between AA and CRC patients, including the differences in composition, the correlation between different viruses and different bacteria, and the differences in SNPs, and to screen the best way to identify CRC, thus providing an alternative certification scheme for AA and CRC.

## RESULTS

### Basic gut microbiome characteristics of AA and CRC patients.

**FIG 1:**
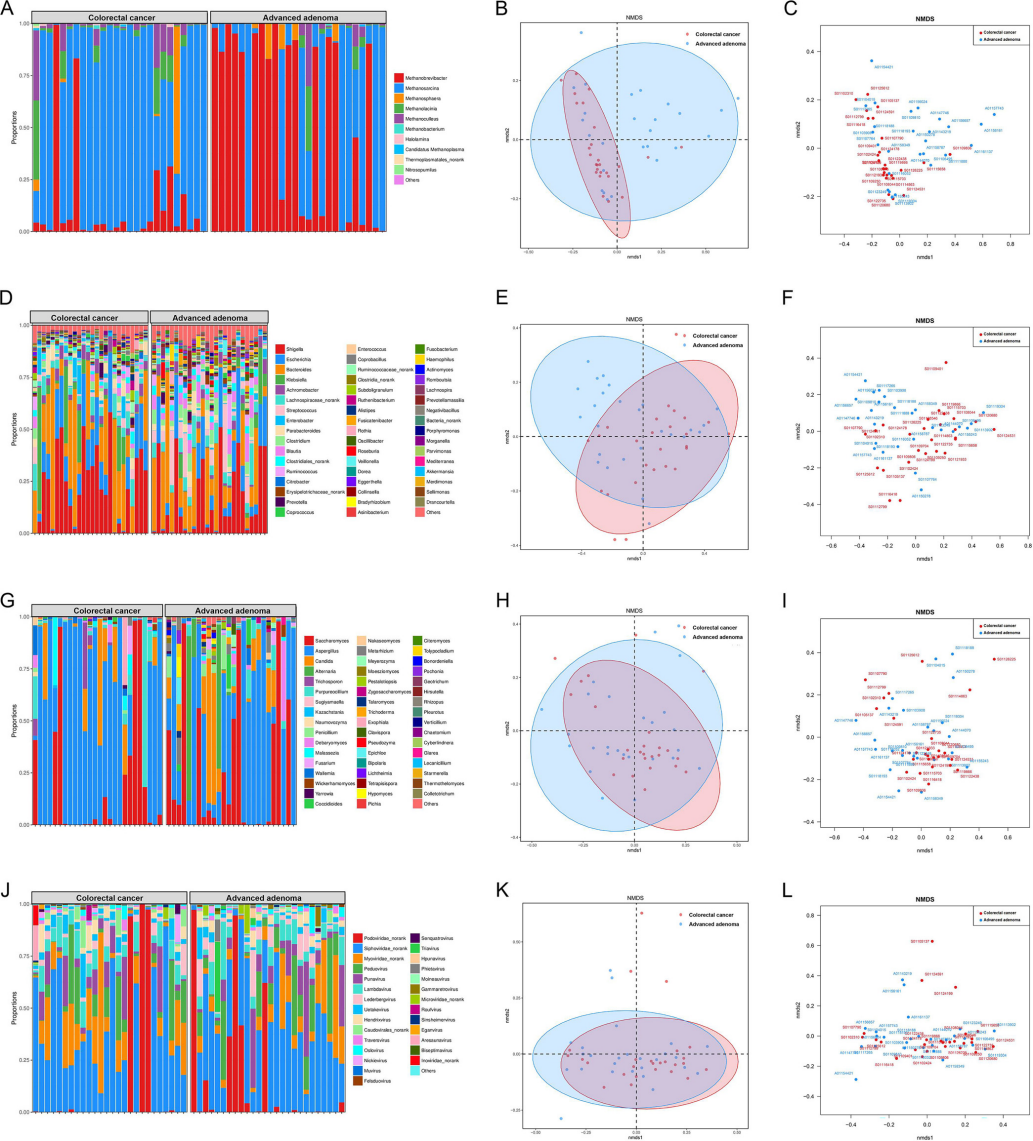
Relative abundance of gut microbiome between AA and CRC patients. The taxonomic assignment of metagenomic sequencing (A) and NMDA analysis (B, C) at the genus level of archaea; the taxonomic assignment of metagenomic sequencing (D) and NMDA analysis (E, F) at the genus level of bacteria; the taxonomic assignment of metagenomic sequencing (G) and NMDA analysis (H, I) at the genus level of fungi; and the taxonomic assignment of metagenomic sequencing (J) and NMDA analysis (K, L) at the genus level of virus.

First, metagenomics sequencing was utilized to test the composition of the gut microbiome of 26 AA patients and 26 CRC patients. As is evident in the taxonomic assignment of metagenomic sequencing, the results illustrated that the composition of the gut bacteria in the two groups of patients was different. For example, at the genus level of archaea, *Methanobrevibacter* and *Methanosarcina* were the two major archaea in both groups. However, the abundance of *Methanobrevibacter* was decreased and *Methanosarcina* was increased in CRC patients compared with those in AA patients (Fig. 1A). Furthermore, regarding bacteria, there were many types of bacteria in patients with the two diseases, and more than 50 types of bacteria were detected in AA and CRC patients, among which the top 5 were *Shigella*, Escherichia, *Bacteroides*, Klebsiella, *and Achromobacter* (Fig. 1D). As shown in Fig. 1D, the abundance of *Shigella* in the CRC group was higher than that in the AA group.

Although less bacteria could be found, there were many kinds of fungi, of which up to 30 types were detected, among which the top 5 were *Saccharomyces*, Aspergillus, *Candida*, *Alternaria*, *and Trichosporon*; and at the genus level of fungi, the abundance of Aspergillus in CRC patients was greatly increased (Fig. 1G). There were approximately 30 species of virus in both patients, among which the top 5 were *Podoviridae*, *Siphoviridae*, *Myoviridae*, *Peduovirus*, *and Punavirus*. At the genus level of the virus, compared with gut polyp patients, *Siphoviridae* appeared more abundant in CRC patients (Fig. 1J).

The nonmetric multidimensional scaling (NMDS) analysis for archaea and bacteria indicated some level of variation between AA and CRC patients, especially the archaeal level (Fig. 1B, C) and bacterial level (Fig. 1E and F). However, NMDS showed no strong clustering between AA and CRC patients at the fungal level (Fig. 1H and I) and viral level (Fig. 1K and L), but some degrees of differentiation were observed between the two groups.

### Differential microorganisms between CRC and AA patients.

To further detect the differences in gut microbiomes between CRC and AA patients, linear discriminant analysis effect size (LEfSe) was used to discover high-dimensional biomarkers, aiming to reveal genomic characteristics. LEfSe used linear discriminant analysis (LDA) to estimate the impacts of the abundance of each component (species) on different effects. Afterward, according to the taxonomic composition, LDA analysis was performed on the samples on the basis of different grouping conditions to determine the communities or species that had significant differences in the division of the samples.

**FIG 2:**
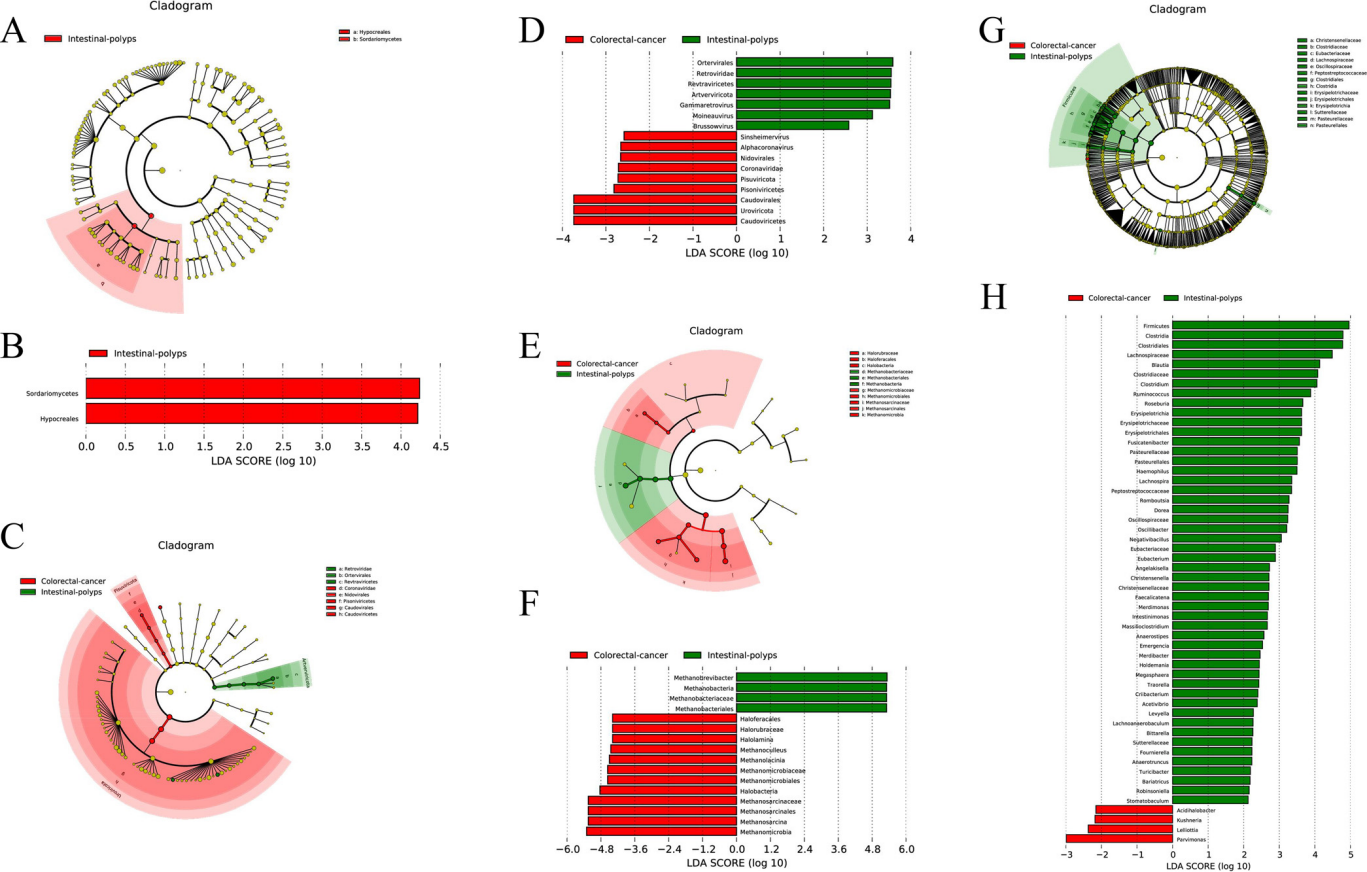
LEfSe analysis filtered out the biomarkers of the microbial community between AA and CRC patients. Cladogram plot of LEfSe analysis (A) and histogram of LDA analysis (B) of fungi; cladogram plot of LEfSe analysis (C) and histogram of LDA analysis (D) of virus; cladogram plot of LEfSe analysis (E) and histogram of LDA analysis (F) of archaea; and cladogram plot of LEfSe analysis (G) and histogram of LDA analysis (H) of bacteria.

Interestingly, no biomarker of fungi in CRC patients was detect, but *Hypocreales* in *Sordariomycetes* was increased in AA patients (Fig. 2A and B). Regarding viruses, *Alphacoronavirus* and *Sinsheimervirus* were upregulated in CRC patients, and *Gammaretrovirus* in *Artverviricota* was increased in AA patients (Fig. 2C and D). Additionally, 16 archaea showed significant differences in the gut microbes between AA and CRC patients, among which *Methanomicrobia* and *Halobacteria* were increased in CRC patients, including *Methanosarcina*, *Methanoculleus*, and *Methanolacinia* at the genus level of *Methanomicrobia* and *Halolamina* of *Halobacteria* (Fig. 2E and F).

Finally, it was found that 54 bacteria showed significant differences in the gut microbiome between AA patients and CRC patients, which was the most abundant group in the four microbial populations. In the study, the AA group was associated with the increased relative abundances of *Firmicutes* at the phylum level and *Clostridia*, *Blautia*, *Clostridium*, and *Ruminococcus* at the genus level. In contrast, the increase in *Acidihalobacter* and *Kushneria* at the genus level was associated with the CRC group (Fig. 2G and H).

### Correlation analysis of microbiomes between CRC and AA patients.

**FIG 3:**
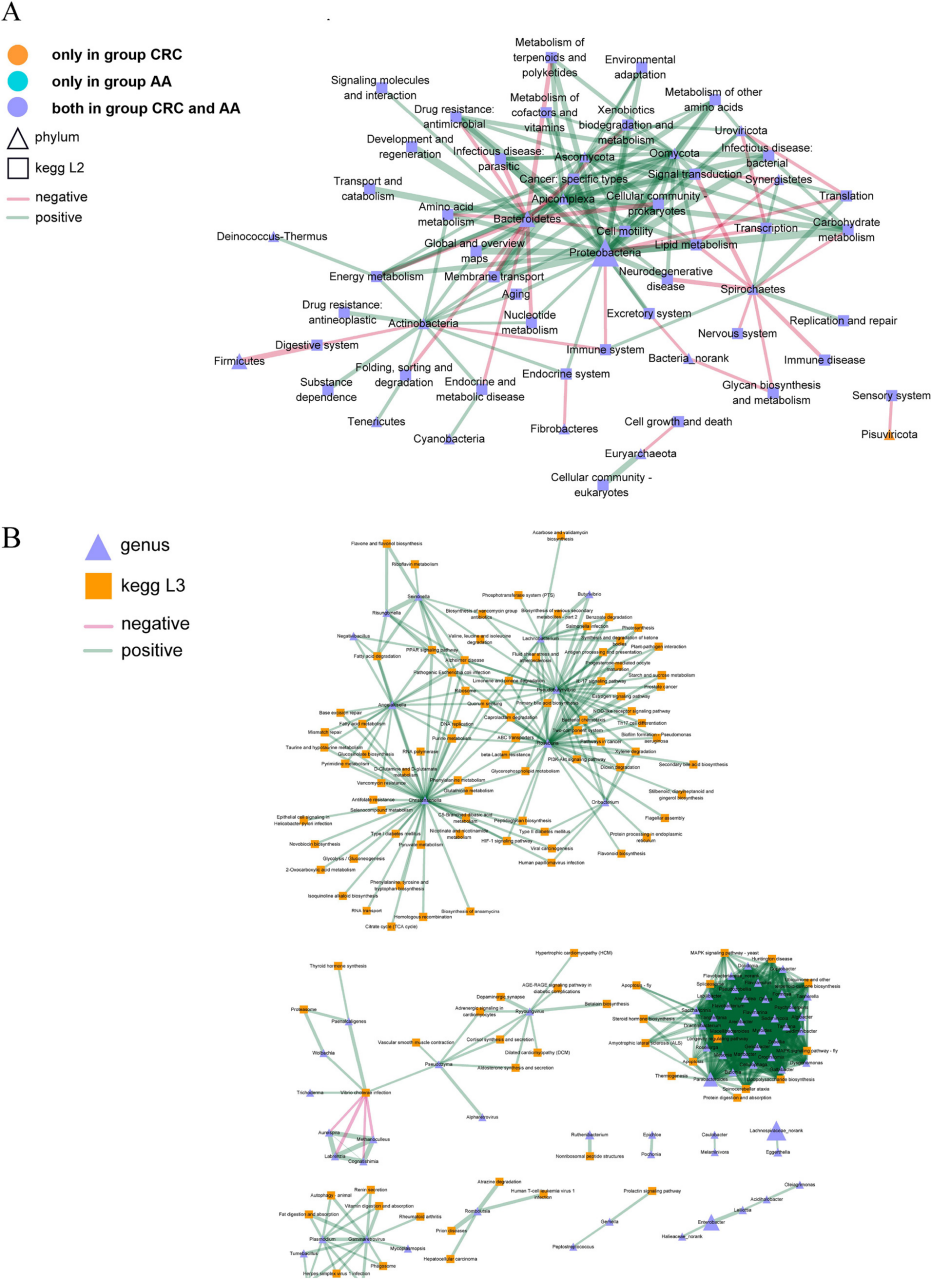
Correlation analysis of the microorganisms and KEGG pathways between the AA and CRC groups. At the phylum level (A), square nodes represent KEGG L2 functions, triangle nodes represent phylum-level species, node size represents abundance, and different colors represent functions and species. The green line indicates a positive correlation, and the red line indicates a negative correlation. The thicker the line is, the higher the correlation is. In addition, at the genus level (B), square nodes represent KEGG L3 functions, triangle nodes represent genus level species, and different colors represent functions and species. The green line shows a positive correlation, and the red line shows a negative correlation. The thicker the line is, the higher the correlation is.

To further explore which microorganisms are more associated with CRC or AA, the correlations between microbiomes and the KEGG pathway of AA and CRC groups were analyzed as previously described and shown in Fig. 3A. Then, at the phylum level, the interactions of the microbiome in the AA and CRC groups with various pathways at the phylum level were analyzed, and it was found that the function of *Pisuviricota* in the CRC group was correlated mainly with the sensory system that may correlate with pain during cancer. *Proteobacteria* in both groups were negatively connected with the immune system, which indicated the inhibition of immunity and was also positively associated with numerous pathways, such as the mitogen-activated protein kinase (MAPK) signaling pathway, longevity regulating pathway, and steroid hormone biosynthesis signaling pathway (Fig. 3A).

Afterward, the interaction between bacteria at the genus level and the interaction with pathways were analyzed. The results confirmed that *Christensenella*, *Angelakisella*, *Pseudobutyrivibrio*, *Roseburia*, and *Lachnobacterium* of bacteria were positively correlated with the pathways of DNA replication, ribosome, glutathione metabolism, and glycerophospholipid metabolism. However, *Aureispira*, *Labrenzia*, *Cogntishimia*, and *Methanoculleus* were negatively connected with Vibrio cholerae infection (Fig. 3B).

### Correlation analysis between microbes and virus.

**FIG 4:**
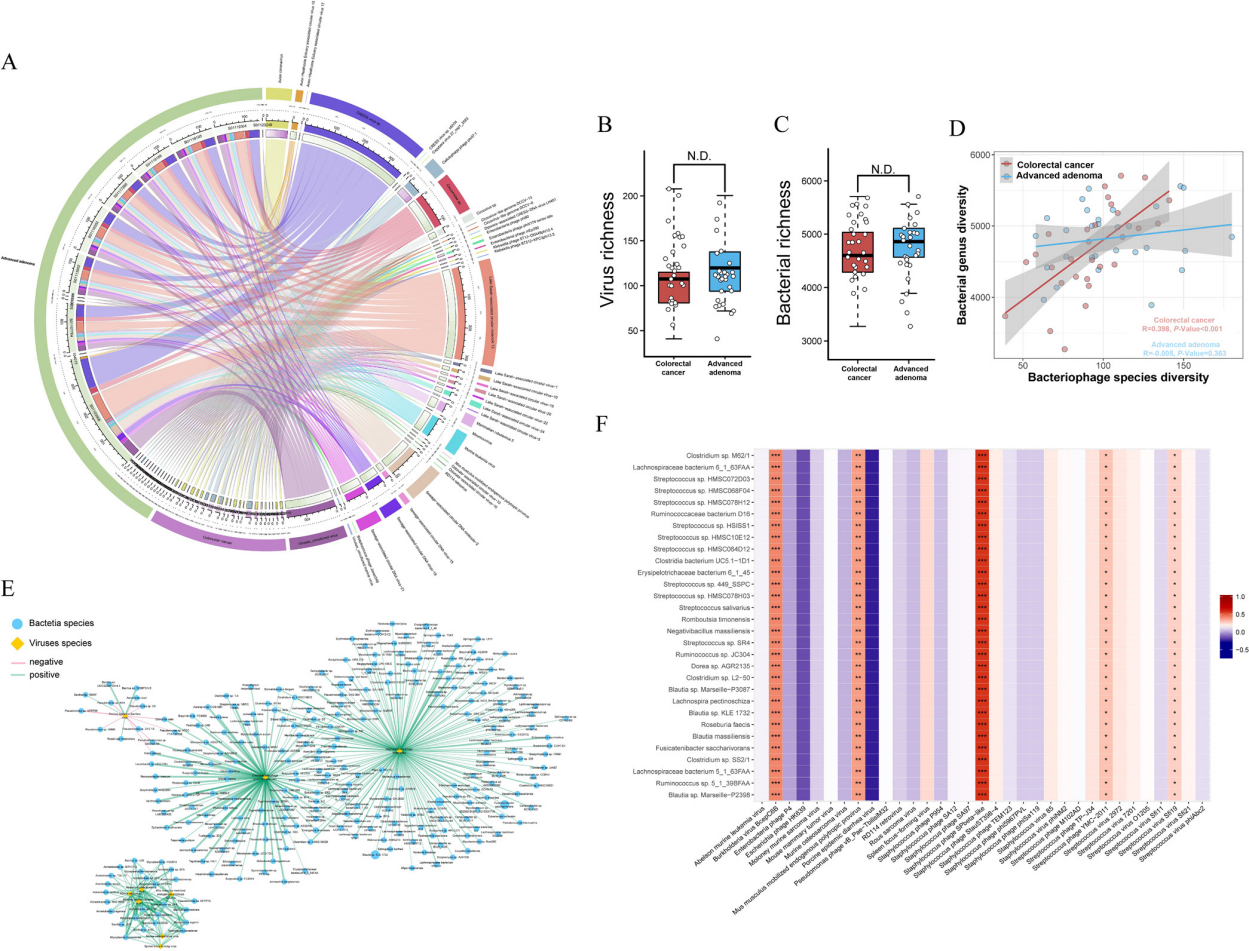
Differential virus analysis between CRC and AA patients. Significant difference analysis in virus diversity (A) and bacterial community richness (B) between CRC and AA patients. The corresponding relationship between CRC and AA groups and species at the virus species level and the proportion of different species in each group and the proportion of each group in different species (C). In the correlation heatmap between differential viruses and differential bacteria (D), *x* and *y* axes are the 11 PathSeq differential viruses and the top 30 differential bacteria in abundance, respectively. The values are shown in different colors, and the legend on the right is the color range of different R values. In the correlation network between differential viruses and differential bacteria (E), circular nodes represent differential bacteria, rhombus nodes represent differential viruses, different colors are used to distinguish between differential viruses and differential bacteria, and the size of nodes represents the abundance. The green line indicates a positive correlation, and the red line indicates a negative correlation. The thicker the line is, the higher the correlation is.

Because of the specialty of phages in regulating the gut microbes, we wondered about the role of phages in regulating AA and CRC. Therefore, the differential viruses in the two groups were analyzed. The results revealed that *CRESS virus* sp., *Circoviridae* sp., and *Lake Sarah−associated circular molecule 12* were more correlated with AA patients and *Cellulophaga phage phi47:1* and *Avian coronavirus* were correlated mainly with the CRC group (Fig. 4A). Furthermore, both groups were associated with other types of microorganisms, which is consistent with previous results.

To further identify the biomarkers associated with the gut microbiome in CRC patients and AA patients, we wondered whether the diversity of viruses or bacteria influenced the progression of CRC, so we tested whether there was a significant difference in virus and bacterial diversity between groups. However, there were no significant differences in the diversity of viruses and bacteria. (Fig. 4B and C). The results suggested that it was not the differences in the diversity of viruses and bacteria that affected the progress of the two diseases (Fig. 4D).

The network of the interaction between bacteria and viruses also confirmed this conclusion (Fig. 4E). Additionally, the top 30 bacteria in terms of the abundance of different viruses were calculated to confirm the biomarkers between the CRC group and AA group (Fig. 4F). The data demonstrated that *Burkholderia virus BcepC6B*, Mus musculus *mobilized endogenous polytropic provirus*, Staphylococcus *phage SPbeta-like*, Staphylococcus *phage YMC-2011*, and Staphylococcus *virus Sfj19* were positively correlated with differential bacteria. In contrast, the increase in other differential viruses may be related to the increase in differential bacteria in the CRC group.

### Prediction model of occurrence and development risk associated with CRC patients, and difference-based phage construction model to distinguish CRC and AA.

**FIG 5:**
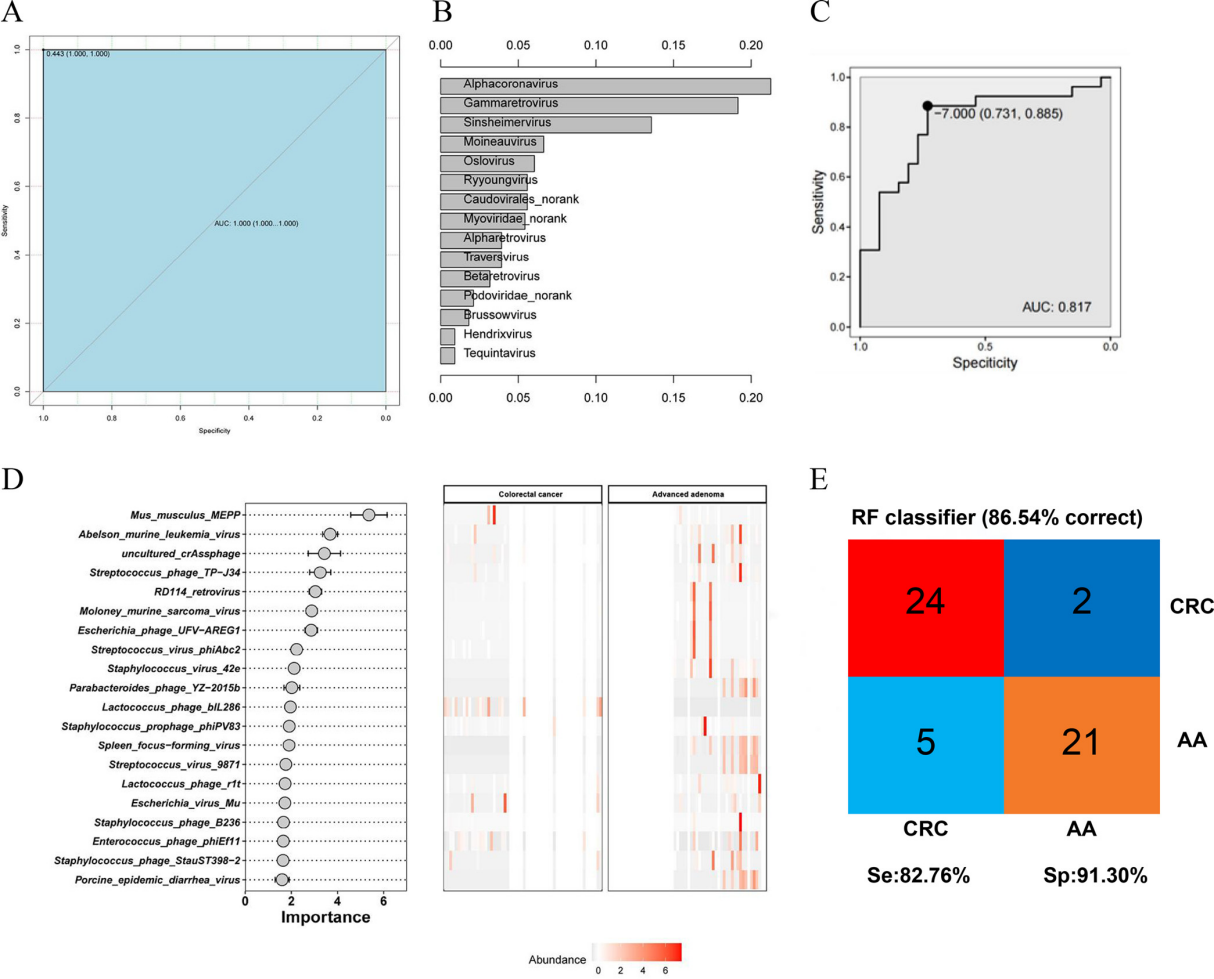
RF model predicted for CRC occurrence. ROC curves of RF (A). Based on ranking, the top 15 viruses were selected for predictive model construction (B). The ROC curve assesses the effectiveness of key marker viruses in disease diagnosis (C). RF identifies key viruses for disease differentiation (D). Evaluation diagram of the RF model (E).

After we identified biomarkers for both groups of diseases, six prediction models of the composition of the bacteriophages and the occurrence risk of CRC were constructed. Among these six prediction models, an RF model based on 15 key viruses showed the optimal classification performance for identifying CRC and AA (Fig. 5A). In the prediction model, the top 15 viruses in terms of importance are displayed in Fig. 5B. The receiver operating characteristic (ROC) curve also evaluated the effects of the key marker virus on the diagnosis of CRC, with an area under the concentration-time curve (AUC) of 0.817, which indicated that these biomarkers may accurately predict the diagnosis of CRC (Fig. 5C). Figure 5D showed the importance of the 20 key viruses to sample differentiation and their changing abundances in different samples, including Mus musculus *MEPP*, *Abelson murine leukemia virus*, and Streptococcus *phage TP−J34*. The accuracy of the results of this set of data was 86.54% in the classification of samples (Fig. 5E). Additionally, the sensitivity and specificity were 82.76% and 91.30%, respectively.

Furthermore, prediction models of the occurrence risk of CRC based on differential archaea and differential bacteria were constructed, respectively. The results proved that the AUC of the model based on archaea was 0.726; the specificity and sensitivity were 0.615 and 0.885, respectively; and the accuracy was 75% (see Fig. S1A to C in the supplemental material). The AUC of the model based on bacteria was 0.646; the specificity and sensitivity were 0.462 and 0.885, respectively; and the accuracy was 78.85% (Fig. S1D to F). The top 19 archaea and the top 20 bacteria of importance in the prediction model are shown in Fig. S1C and Fig. S1F, respectively. Compared with the models based on bacteriophages, the accuracy of the models based on differential archaea and bacteria was lower.

### SNP analysis between AA and CRC groups.

**FIG 6:**
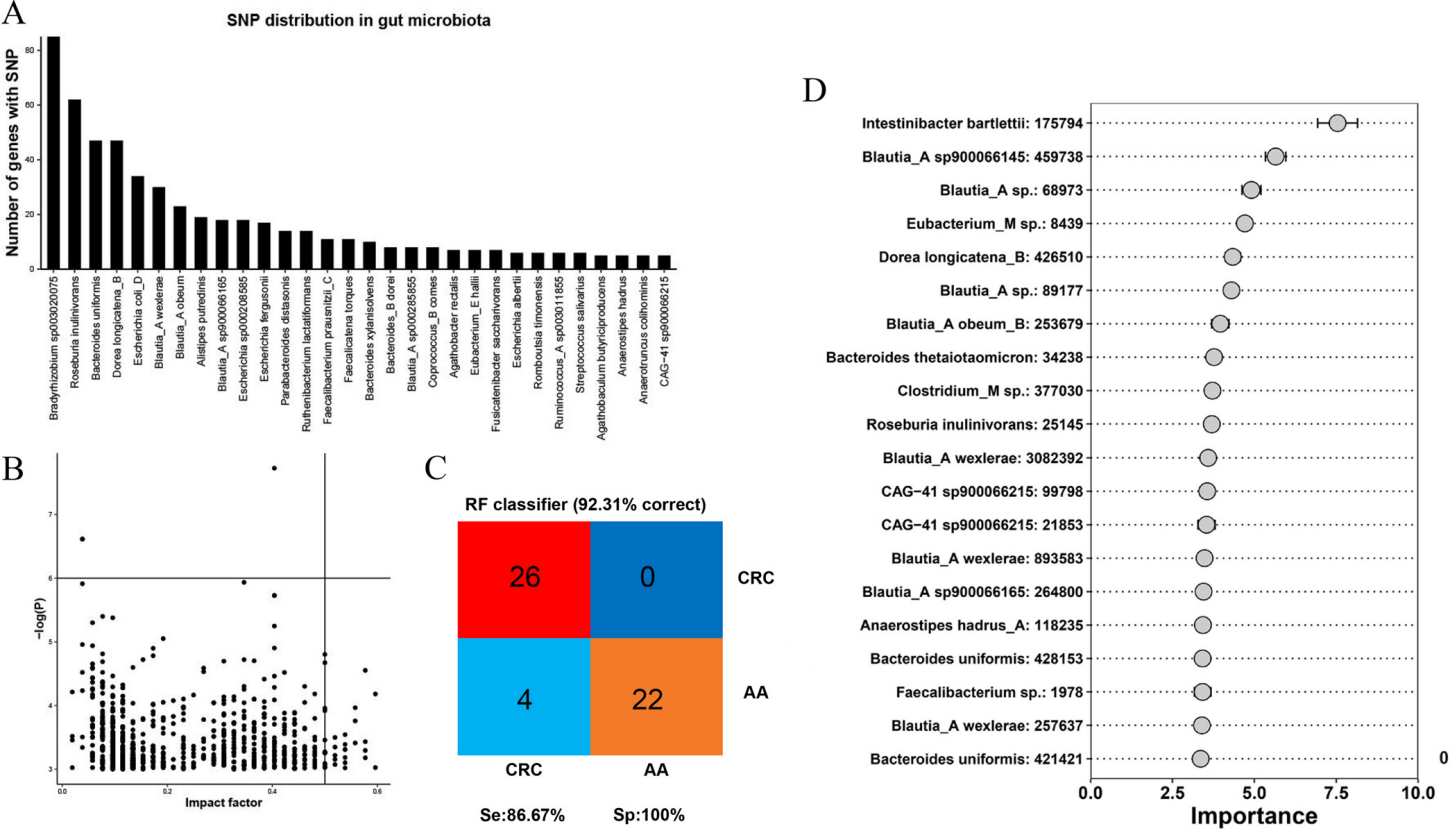
Analysis of SNPs between AA and CRC patients. Bar chart of the number of species SNP genes (A). Scatter diagram of the impact factor index for SNP site. The impact factor is used as the horizontal coordinate, and the *P* value of the SNPs is used as the vertical coordinate (B). Evaluation diagram of the RF model (C). RF identifies key SNPs for disease differentiation (D).

The number of genes associated with SNP was calculated at the genus and species levels to identify the main gut microbe species with SNPs, and the top 30 gut bacterial genera or species with the highest number of SNPs were selected, as shown in Fig. 6A. The higher the bar chart is, the more nonsynonymous SNP genes in the microbe. Then, the impact factor index of each identified nonsynonymous SNP was calculated to show the possibility of a target for CRC prediction (Fig. 6B). The abscissa of Fig. 6B represents the impact value of each species, and the ordinate represents the significance corresponding to the impact value. The higher the *y* value is, the greater the correlation is with the group. The closer the origin of the SNP is to the upper-right corner of the image, the more likely it is to be used as a disease prediction target. Additionally, by recognizing key SNP sites, the importance of each SNP site was obtained through the RF model, and the accuracy in the classification of samples was 92.31% (Fig. 6C). Besides, the sensitivity and specificity were 86.67% and 100%, respectively, which indicated that these selected biomarkers may accurately predict the diagnosis of CRC. The key SNP site is displayed in Fig. 6D as a candidate disease prediction target.

## DISCUSSION

Most CRC is caused by AA, so analyzing the differences in gut microbes between AA and CRC patients under pathological conditions may provide more useful key biomarkers for the prediction of disease. However, there is still no acknowledged biomarkers to distinguish AA and CRC. To find an effective microbial target from bacteriophages and microbial SNPs, the gut microbiome of the profiles of AA patients and patients with CRC was compared by metagenomic analysis. Although recent studies have investigated gut microbial dysbiosis in CRC patients and reported diagnostic potential using metagenomic sequencing (22, 23), these promising results did not identify which gut microbiome or their characteristics changed during the progression from AA to CRC.

In the study, the structure and diversity of gut microbiota between CRC and AA were analyzed, and it was found that the abundance of *Shigella* in the CRC group was higher, which was the same as the results reported by Wang Tingting et al (24). They analyzed fecal bacterial diversity in CRC patients (*n* = 46) and healthy volunteers (*n* = 56) by 454 pyrosequencing of the V3 region of the 16S rRNA gene. The results showed that the abundances of 11 intestinal flora, including *Enterococcus*, Escherichia*/Shigella*, and *Enterococcus*, were significantly enriched in CRC patients. *Shigella* is a group of short Gram-negative bacilli. After the invasion of intestinal mucosal epithelial cells and lamina propria, it caused an inflammatory response and small vascular circulation disorders, thus leading to intestinal mucosal inflammation, necrosis, and ulceration, which can cause diarrhea (25). The lesions involved mainly the rectum and sigmoid colon, and in severe cases, the whole colon and terminal ileum could be involved. The recurrence of intestinal mucosal inflammation may be the cause of CRC. *Shigella* can participate in intestinal inflammation by producing toxins. For example, Shiga toxin (Stx) and Shiga-like toxin (SLT) are related protein toxins produced by Shigella dysenteriae and certain strains of Escherichia coli (26). Shiga toxin binds specifically to the receptor Gb3 on the host cell membrane and is transported retrograde into the trans-Golgi network and endoplasmic reticulum (ER). Shiga toxin subunit A targets the ribosome and inactivates protein synthesis through its 28S RNA-specific *N*-glycosidase activity after entry from the ER into cytoplasm as part of the ribosomal elongation complex (27). Due to the action of the toxin, the intestinal mucosa appears to undergo epithelial cell death, submucosal inflammation, and capillary thrombosis and even necrosis, exfoliation, the formation of ulcers, and eventually tumorigenesis (28). Under normal circumstances, the intestinal flora is in a state of balance. Once the intestinal flora is disordered, the intestinal microecological balance would be broken, which induces CRC. *Shigella* may cause CRC by affecting other intestinal bacteria, such as Escherichia coli, Bacteroides fragilis, and Enterococcus faecalis (29). Besides, the changes in the number of other gut microbiota can also cause changes in *Shigella*, which may also be the cause of CRC.

In this study, it was found that the abundance of *Shigella* was differentially expressed between CRC and AA groups, but the regulatory mechanism behind this differential expression is still unclear. Therefore, further studies should be carried out to explore the underlying mechanisms.

In this study, a sample of people from the same region with the same diet and lifestyle were included. The relative abundance of each type of gut microbiome in AA and CRC patients was investigated first, and the biomarkers of the gut microbiome between AA and CRC patients were filtered by LEfSe analysis. Then, the correlation between gut microbiome and KEGG pathway in the AA group and CRC group was analyzed, and the microbiome interactions of different pathways both groups were analyzed at the phylum and genus levels, which revealed the different effects and that different types of microbiomes may play important roles in AA and CRC.

However, there is still an important problem to be solved; the composition of the gut microbiome is too complex to analyze. It deserves to be mentioned that a machine-learning algorithm, the most advanced artificial intelligence solutions for predicting the occurrence of CRC, is a tool to make analysis possible. Before we constructed the disease prediction model, the differences and correlations between bacteria and bacteriophages in the CRC group and AA group were analyzed. According to the results, the diversity of different bacteria and phages was consistent between the two groups, and there was a correlation between different bacteria and different phages. Bacteriophages are a type of virus, and bacteria are a type of prokaryote. To be biologically active, bacteriophages must parasitize bacteria. Considering the large size of the bacterial community, it was not easy to analyze and construct a model. Based on bacteriophage biological characteristics and the results of the previous steps, bacteriophages were used to construct the prediction model, which was significantly associated with bacteria.

Moreover, the progress of sequencing technology and the generation of big data also provide the possibility for us to search for biomarkers and predict the occurrence of diseases, and the mega data algorithm has a higher accuracy (23, 30). At present, algorithms based on mega data have been used in the prevention and monitoring of hypertension, diabetes, tumors, and other diseases. The progress of the algorithm could promote the accuracy of tumor prevention, diagnosis, and prognosis monitoring. As shown in this study, a large number of bacteria and viruses were involved, and the interaction between them is extremely complicated, which is difficult to achieve in basic research (26, 31).

Within the context of microbial interactions, how gut bacterial species might interact with bacteriophages was shown. Our analysis indicated that the bacteriophage community may play a different regulatory role in the survival of microbiome Streptococcus species in the diseased gut compared with the AA group. The study displayed that *Burkholderia virus BcepC6B*, Mus musculus *mobilized endogenous polytropic provirus*, Staphylococcus *phage SPbeta-like*, Staphylococcus *phage YMC-2011*, and Staphylococcus *virus Sfj19* were positively correlated with differential bacteria. Mus musculus *mobilized endogenous polytropic provirus* is a virus of eukaryotic animal origin. Current studies confirmed that Mus musculus *papillomavirus 1* (MmuPV1) in such viruses was associated with the induction of immunosuppressive skin cancer (32). A taxonomically correlated pair of bacterial and viral features may signal events of genomic integration, while this observation could also be interpreted as a form of microbiome interactions. Our analysis was focused on the members of the enteric DNA virome to inspect how they interact with their bacterial counterparts; however, the variations might not be explained by clinical factors alone but may also be associated with many other lifestyle factors in clinical microbiome studies. Whether the change of enterovirus abundance is the cause or result of AA progression to CRC is a direction worthy of further study. These different bacteriophages may provide novel insights into how to prevent benign AA from progressing into malignant CRC.

Chen et al. (33) demonstrated that the differences in SNPs in the gut microbes within individuals were significantly smaller than those between individuals. By analyzing the SNP in gut microbes of AA and CRC, we were surprised to find the significant differences in SNPs between the two diseases at the base level. Although the results of our study are different from those of Xiao et al. (21), it was confirmed that SNPs with the ability to maintain stability have a huge advantage in distinguishing AA and CRC. In the future, the SNP is expected to be a predictive target for distinguishing CRC from AA, which provides an alternative for differentiating the two diseases. In this study, the RF model based on 15 key viruses showed 86.54% of accuracy in the classification of CRC and AA. Additionally, by recognizing key SNP sites, the importance of each SNP site was obtained through the RF model, and the accuracy in the classification of samples was 92.31%. Microbial SNPs showed a higher performance in identifying CRC, and it was little affected by environmental factors and had little change over the years (13), which may provide a new research direction for preventing the progression of AA and CRC.

However, this study still has some limitations. First, only 26 CRC cases and 26 AA controls were recruited. Due to the insufficiency of the sample size, there was a lack of validation set data in the construction of the disease prediction model, which might cause the problem of model overfitting. The accuracy of the model in this study needs to be improved with the subsequent increase in the number of samples. Second, an insufficient number of healthy individuals was the weakness of this study. A large number of studies have reported the differences of gut microbiota between healthy people and CRC patients and between healthy people and AA patients. However, there are relatively few studies on the screening of microbial targets for the identification of AA and CRC. Therefore, healthy people were not included for metagenomic sequencing in the design of this study. Undoubtedly, adding a healthy control group would make the findings more convincing. Third, the lack of in-depth molecular mechanism research is another important drawback of this study. However, metagenomic sequencing data were used to analyze the differences in microbial abundance and SNP characteristics between CRC and AA patients and to describe the microbial profiles of CRC and AA patients. It can provide data support for the molecular mechanism study on the mechanism of AA conversion to CRC and the pathogenesis of CRC. In subsequent large-scale multicenter clinical studies, the samples from healthy people can be recruited to further validate the results of this study. Subsequently, continuous follow-up of AA patients and regular confirmation of gut microbiota changes during the progression of AA to CRC patients would be meaningful and worthy additions to a future study.

CRC is one of the most common malignant tumors with a high incidence, and AA has a high cancerization progression rate to CRC. Researchers seeking biological targets that distinguish AA from CRC are expected to provide novel targets for the prevention of CRC and new research directions for the study on the pathogenesis of CRC. At present, we aimed to explore microbial targets that could identify AA and CRC from a microecological perspective. Metagenomic sequencing data of stool samples from AA and CRC patients were analyzed to map the microbial community composition of these patients. Potential microbial targets, including bacteria, fungi, and bacteriophages that can be used to identify AA and CRC, were screened by differential analysis. The interaction network between microorganisms and host was constructed. It was also found that the accuracy of a prediction model based on microbial SNPs was higher than that of the gut microbiome. Although further multicenter large-sample clinical studies and molecular mechanism studies are needed to verify the results and the accuracy of the model, this study could provide a new perspective and method for identifying AA and CRC from the microbial perspective.

## MATERIALS AND METHODS

### Subjects.

Patients with CRC and AA at Huzhou Central Hospital from March 2020 to February 2021 were studied. On the other hand, CRC and AA were confirmed by pathological diagnosis, and the clinical stages were determined according to the American Joint Committee on Cancer (AJCC) staging guidelines. The clinical protocols involving the patients and the informed consent form were approved by the Ethics Committee of Huzhou Central Hospital (no. 20191101-02) and Chinese Clinical Trial Registry (http://www.chictr.org.cn; ChiCTR2000034061).

A total of 26 patients with CRC had moderately differentiated adenocarcinomas. Moreover, these CRC patients were all MicroSatellite stability (MSS) type, without RAS mutation, and had pathological stage II or III. The adenomas of all AA patients were larger than 1 cm and belonged to tubular adenomas. The inclusion criteria are as follows: CRC patients and progressive adenoma were diagnosed by pathological examination and volunteered to participate in the study.

The exclusion criteria are as follows: (i) patients with other primary cancers; (ii) patients with other gut diseases, such as ulcerative colitis and Crohn’s disease; (iii) patients with a medicine history of oral microbial agents and lipid-regulatory agents within the last 2 months; and (iv) patients with known primary organ failure. All subjects signed informed consent under the guidelines approved by the Ethics Committee of Huzhou Central Hospital.

### Collection of clinical data and stool samples.

Basic patient information, clinical indicators, and pathological data were obtained from the medical record management system of Huzhou Central Hospital with informed consent from patients. Stool samples were collected in the morning prior to breakfast. Approximate 5- to 10-g stool samples were obtained after defecation without the use of a purgative or lubricant. Within half an hour, the stool samples were stored in an ultralow temperature freezer. The sample preservation time was not beyond 1 month. Finally, 52 stool samples from AA patients and CRC patients collected from March 2020 to February 2021 were analyzed after the patients signed informed consent forms, and unqualified specimens were eliminated as well. The basic characteristics of these patients are shown in Table 1. There were no differences in diet and lifestyle among all samples.

**TABLE 1:** Characteristics of study participants at admission

Characteristic	Value^*a*^ for patients with:		*P* value
	Colorectal cancer	Advanced adenoma	
Cases	26	26	
Male	14	17	0.40
Age	65.58 ± 8.39^*b*^	60.27 ± 11.32^*b*^	0.06
Current smoker	6	8	0.53
Current drinking	8	5	0.26
Known diabetes	4	3	0.69
Known hypertension	3	2	0.64
Inflammatory bowel disease	0	1	0.31
Family history	1	0	0.31

a Values are *n* unless otherwise noted.

b Values are mean ± SD yrs.

### Metagenomics sequencing.

Fecal samples were resuspended in phosphate-buffered saline and sequentially filtered using a 0.8-μm (polyethersulfone [PES]) filter (Sartorius). Any remaining DNA that was not encapsidated was degraded by treatment with a mixture of benzonase and micrococcal nuclease (New England BioLabs), followed by EDTA inactivation of DNases. The remaining supernatant was subjected to lysis, and viral DNA was extracted using the QIAamp viral RNA minikit without carrier RNA (Qiagen). Metagenomic shotgun sequencing libraries were constructed and sequenced at Shanghai Biozeron Biological Technology Co. Ltd. In brief, for each sample, a Nextera XT DNA library preparation kit from Illumina was used to construct sequencing libraries, and the concentration of all libraries was measured by a high-sensitivity double-stranded DNA kit on a Qubit Fluorometer (Thermo Fisher Scientific). All samples were sequenced in an Illumina Novoseq instrument with paired-end 150-bp (PE150) mode. The amount of data in each sample was close to 10 G. Raw sequence reads underwent quality trimming using Trimmomatic (http://www.usadellab.org/cms/?page=trimmomatic) to remove adaptor contaminants and low-quality reads (34). Reads run through quality control were then mapped against a mouse genome (NCBI) by the BWA-MEM algorithm (parameters: -M -k 32 -t 16; http://bio-bwa.sourceforge.net/bwa.shtml). After removal of host-genome contaminations and low-quality data, reads were called as clean reads and used for further analysis.

The taxonomy of clean reads for each sample was determined by the PathSeq pipeline distributed in GATK v4.1.3 (35) using the default database downloaded from Broad Institute (parameters: –min-score-identity = 0.90 and –identity-margin = 0.02). By default, PathSeq will discard alignments if both read pairs do not match the same organism. The taxonomy database included all bacterial, archaeal, fungus, and virus genome sequences in the NCBI RefSeq database. All reads were classified to seven phylogenetic levels (domain, phylum, class, order, family, genus, and species) or were unclassified. The relative abundance of a certain level is a total of abundance of species belonging to that level. According to the annotations generated by PathSeq, the relationship between host genome and phage was constructed.

### SNP analysis.

The UHGG database was used as the reference genome, BWA (MEM-T 20-M) was used to compare the sequenced fragments back to the reference genome, the Tassel GLM model was used for correlation analysis with phenotype, and 4,644 metagenome-assembled genomes (MAGs) were used as reference genomes for analysis in this study. The number of genes associated with a nonsynonymous single nucleotide variant (SNV) was calculated at the genus and species levels to identify the main gut microbe species with SNPs.

First, the impact factor index of each identified nonsynonymous SNP locus was calculated. The index was calculated as the ratio of the number of bases of this gene locus to the number of predicted bases in the group in all research samples, and it can reflect the consistency of a SNP in different individuals to a certain extent. The stronger the consistency, the higher the probability of the site as a target site for disease prediction.

### Multiple machine-learning models for evaluating the predictive ability of SNPs.

The methods of model construction were as described previously (36). First, before model construction, the recursive feature elimination (RFE) algorithm based on the sklearn.feature_selection method was applied to feature selection. Then, random forest (RF) and gradient boosting decision tree (GBDT) machine-learning models were based on sklearn.ensemble. Additionally, a support vector machine (SVM) was based on sklearn.svm, and a neural network (NN) was based on sklearn.neural_network. Besides, a CatBoost machine-learning model was constructed using the Catboost package (version 0.16.5). Logistic regression (LR) used relevant functions in the R language rminer package (version 1.4.5) for modeling analysis and importance calculation of variables by using multinom from nnet package.

### Statistical analysis.

Taxonomies were sorted by the levels of phyla, and each phylum was clustered separately by class. The inclusion criterion for plotting features had a minimum absolute magnitude of interkingdom or intrakingdom correlation of 0.6 and a false discovery rate (FDR) of less than 0.05 in either one or both of control and CRC groups. For visualization, the correlations with absolute coefficients below 0.3 were masked to show all signals that were exclusively significant. *P* values less than 0.05 after multiple comparison correction using the FDR method were considered significant. A two-tailed Mann-Whitney U test was used to determine statistically significant differences between cases and controls.

### Data availability.

The data sets generated during the current study are publicly available and can be obtained from the NCBI database (SRP339836).
