# Supplementary material for: Different Characteristics in Gut Microbiome between Advanced Adenoma Patients and Colorectal Cancer Patients by Metagenomic Analysis
Source: Microbiol Spectr. 2022 Dec 1;10(6):e01593-22. doi: 10.1128/spectrum.01593-22 (PMC9769752; doi:10.1128/spectrum.01593-22)

Fig S1. Construction of prediction models according to differential archaea and bacteria.

ROC curves of RF model accroding to differential archaea (A), evaluation diagram of the RF model (B), and the top 19 archaea were selected for predictive model construction basing on ranking (C). ROC curves of RF model accroding to differential bacteria (D), evaluation diagram of the RF model (E), and the top 20 bacteria were selected for predictive model construction basing on ranking (F).

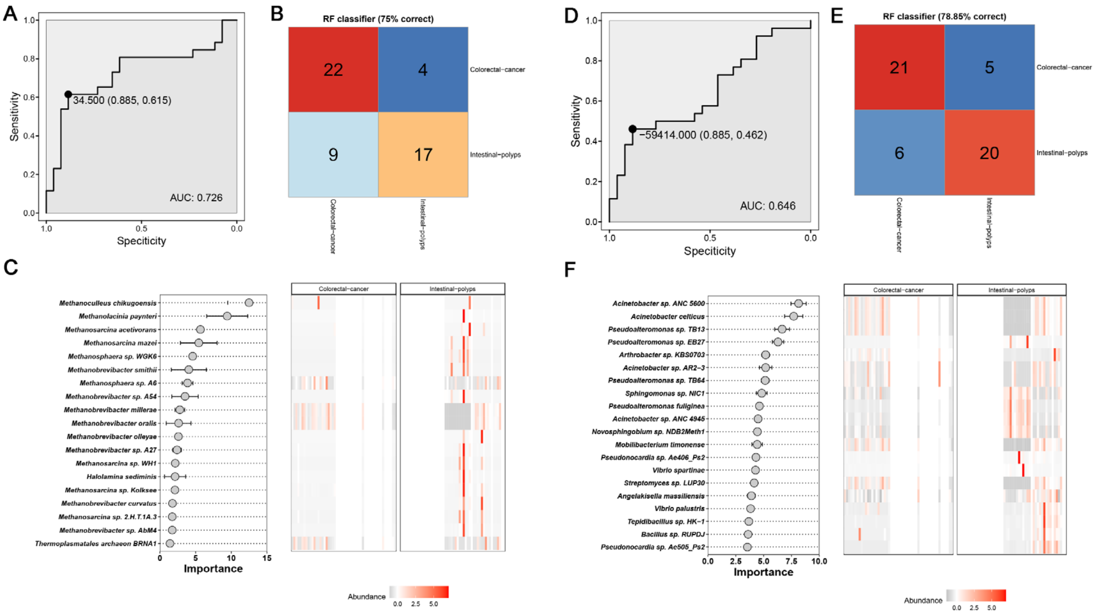

Supplement: Supplemental file 1 — Fig. S1. Download spectrum.01593-22-s0001.pdf, PDF file, 0.6 MB [file spectrum.01593-22-s0001.pdf]
